# On the Reliability of a Solitary Wave Based Transducer to Determine the Characteristics of Some Materials

**DOI:** 10.3390/s16010005

**Published:** 2015-12-23

**Authors:** Wen Deng, Amir Nasrollahi, Piervincenzo Rizzo, Kaiyuan Li

**Affiliations:** 1 School of Automation, Northwestern Polytechnical University, Xi’an 710072, China; dengwen0626@gmail.com; 2 Laboratory for Nondestructive Evaluation and Structural Health Monitoring Studies, Department of Civil and Environmental Engineering, University of Pittsburgh, 3700 O’Hara Street, 729 Benedum Hall, Pittsburgh, PA 15261, USA; amn70@pitt.edu (A.N.); kal126@pitt.edu (K.L.)

**Keywords:** highly nonlinear solitary waves, nondestructive evaluation, modulus of elasticity, concrete, magnetostrictive sensors

## Abstract

In the study presented in this article we investigated the feasibility and the reliability of a transducer design for the nondestructive evaluation (NDE) of the stiffness of structural materials. The NDE method is based on the propagation of highly nonlinear solitary waves (HNSWs) along a one-dimensional chain of spherical particles that is in contact with the material to be assessed. The chain is part of a built-in system designed and assembled to excite and detect HNSWs, and to exploit the dynamic interaction between the particles and the material to be inspected. This interaction influences the time-of-flight and the amplitude of the solitary pulses reflected at the transducer/material interface. The results of this study show that certain features of the waves are dependent on the modulus of elasticity of the material and that the built-in system is reliable. In the future the proposed NDE method may provide a cost-effective tool for the rapid assessment of materials’ modulus.

## 1. Introduction

In the last ten years interest in the use of highly nonlinear solitary waves (HNSWs) in physics and engineering has increased considerably. These waves are compact nondispersive mechanical waves that can form and travel in highly nonlinear systems, such as a closely packed chain of elastically interacting spherical particles [1,2,3] or in the tensegrity units [4,5]. Typically, solitary waves are induced by tapping the first particle of the chain with a particle striker identical to the spheres forming the chain.

In the study presented in this paper, we assembled four built-in systems, hereinafter indicated as the HNSW transducers, to excite and sense HNSWs. Each transducer consisted of a chain of spherical particles surmounted by an electromagnet to lift and release the striker, and a coil and two permanent magnets around the nine-th particle of the chain to sense the propagating wave using the magnetostriction principles. We then quantified the reliability of these transducers at inferring the modulus of a few materials placed in contact with the transducers. The method exploited the dynamic interaction between the solitary waves propagating within the chain and the test specimens.

Two sets of experiments were conducted. In the first one, the four transducers were tested against samples made of soft polyurethane, hard polyurethane, and steel. The cases of free transducers and transducers partially immersed in water were also considered to set a baseline data. The results were compared to assess the transducers repeatability, and were then compared to the numerical predictions obtained with a discrete particle model (DPM) to verify the accuracy of our instruments. The study on the dynamical interaction between solitary waves and bulk materials is not novel; Manciu and Sen [6] investigated the wave reflections from rigid wall boundaries. Falcon *et al.* [7] studied the collision of a column of N beads with a fixed wall. Job *et al.* [8] evaluated the collision of a single solitary wave with elastic walls with various stiffness. Yang *et al.* [9] demonstrated that the formation and propagation of reflected HNSWs depend on the modulus of elasticity and geometry of the adjacent medium. Finally, Ni *et al.* [10] monitored the curing of fresh cement using a HNSW-based transducer. Our group has also investigated the interaction of HNSWs with plates and slender beams [11,12,13,14]. In all the above studies, the sensing element was either a force sensor placed at one end of the chain or a sensor bead, *i.e.*, a piezo-element glued in between two half particles and inserted along the chain. 

In the second investigation, the same approach was used to find the modulus of elasticity of hardened concrete. The findings were then compared to the results obtained from the conventional ultrasonic pulse velocity (UPV) method, and the destructive tests standardized in the ASTM C469, ASTM C39, and the ACI 318 [15,16,17]. The purpose of this second investigation was the development of a novel nondestructive evaluation (NDE) method to estimate the strength of concrete in existing structures.

Many NDE methods for concrete have been proposed, tested, and commercialized in the last three decades. The most common method is perhaps the one based on the measurement of the velocity of linear bulk ultrasonic waves propagating through concrete. Traditionally, commercial transducers are used to generate and detect ultrasonic bulk waves [18,19,20,21,22,23,24]. Parameters, such as wave speed and attenuation are theoretically correlated to the properties of the material. This approach is usually referred to as the UPV method, and it is extensively used in practice and in researches for monitoring the properties of hardened or fresh concrete. When the back wall of the sample is impractical, the wave reflection method is used, and impact-echo and pulse-echo methods are two common used techniques of this kind [25,26,27]. 

With respect to ultrasonic-based NDE, the proposed HNSW-based approach: (1) exploits the propagation of HNSWs confined within the grains; (2) employs a cost-effective transducer; (3) measures different parameters (time of flight, speed, and amplitude of one or two solitary pulses) that can be eventually used to correlate few concrete variables; (4) does not require any knowledge of the sample thickness; (5) does not require an access to the sample’s back-wall. The solitary waves based method may resemble the Schmidt hammer that consists of a spring-driven steel hammer that hits the specimen with a defined energy. Part of the impact energy is absorbed by the plastic deformation of the specimen and transmitted to the specimen, and the remaining impact energy is rebounded. The rebound distance depends on the hardness of the specimen and the conditions of the surface. The harder is the surface, the shorter is the penetration time or depth; as a result, the higher is the rebound [28,29]. The differences between our method and the Schmidt hammer are: the hammer cannot be applied onto fresh concrete and cement; only one parameter, the rebound value, is used in the Schmidt hammer test, while multiple HNSWs features can, in principle, be exploited to assess the condition of the underlying material. The Schmidt hammer may induce plastic deformation or microcracks to the specimen, while the HNSW approach is purely nondestructive as there is not mechanical impact on the material under testing. The present paper is entirely novel with respect to paper [30]. There, one HNSW transducer was used to monitor the curing of one concrete cylinder. The transducer embedded two sensor beads within the chain of particles forming the transducer.

For all the above, the novelty of this paper is are: (1) more transducers were designed, assembled and tested to assess their repeatability; (2) the transducers carried a sensing element based on magnetostriction. This was reported in [30] for the first time in a study that compared three sensing elements: a conventional sensor bead; a magnetostrictive sensing approach; and a piezo-cylinder. However, no other studies considered magnetostriction as a possible tool to detect the solitary waves, and this paper investigates the reliability of magnetostrictive sensing for solitary waves; (3) a hardware system was designed and assembled to drive the transducers simultaneously without the need of manual plug-and-play; (4) hardened concrete was tested in order to investigate the feasibility of this novel NDE method to assess the strength of existing concrete.

## 2. Background 

**Figure 1:**
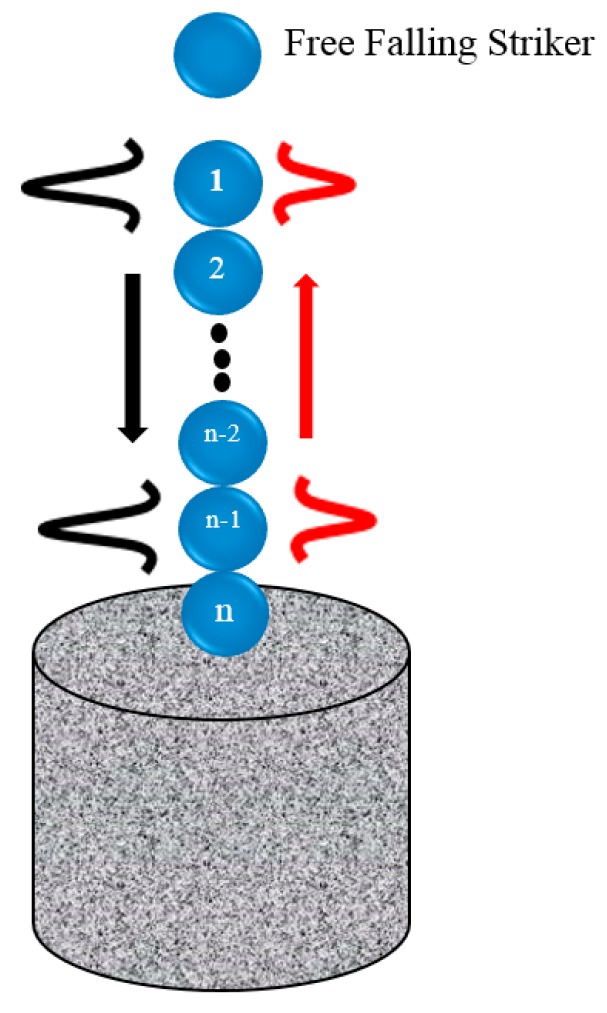
General scheme of the nondestructive evaluation method based on the propagation of highly nonlinear solitary waves.

This section describes the underlying basis of HNSWs, and it follows the symbolism adopted in [10,31], which in turn is based on the analytical formulation provided in several works including [1,3,31]. Figure 1 illustrates the general principles of the proposed method: a 1-D chain of spherical particles is in contact with the material to be assessed; the impact of a striker, *i.e.*, a particle of equal size and mass of the other particles composing the chain, generates a single pulse that propagates through the chain. The interaction between two adjacent beads is governed by the Hertz’s law [32,33]:

(1) $$F=A\delta^{3/2}$$

and it establishes a relationship between the compression force *F* of the granules and the closest approach of the particle centers. In Equation (1) the coefficient *A* is given by:

(2) $$A=\left\{ \begin{array}{l} A_{c}=E\sqrt{2a}/3(1-\nu^{2})\\ A_{w}=\frac{4\sqrt{a}}{3}{\left(\frac{1-\nu^{2}}{E}+\frac{1-{\nu_{w}}^{2}}{E_{w}}\right)}^{-1} \end{array}\right.$$

where *A_c_* refers to the case of particle-to-particle contact, and *A_w_* refers to the case of particle-to-wall (infinite curvature radius) contact case. In Equation (2) *E*, *a*, and *ν* are modulus of elasticity, radius, and Poisson’s ratio of the spherical beads, respectively, whereas *E_w_* and *ν_w_* are the modulus of elasticity and Poisson’s ratio of the wall, respectively.

The combination of the nonlinear interaction (Equation (1)) and a zero tensile strength in the chain of spheres leads to the formation and propagation of compact solitary waves [33]. When the wavelength is much larger than the particles’ diameter, the speed *V_S_* of the waves depends on the maximum dynamic strain *ξ_m_* [33] which, in turn, is related to the maximum force *F_m_* between the particles in the discrete chain [3]. When the chain of beads is under a static pre-compression force *F_0_* (*F_0_ << F_m_*), the initial strain of the system is referred to as *ξ_0_*. It should be noted that in configurations like the one shown in Figure 1, the pre-compression is provided by the self-weight of the chain. The speed *V_s_* has a nonlinear dependence on the normalized maximum strain *ξ_r_* = *ξ_m_*/*ξ_0_*, or on the normalized force *f_r_* = *F_m_*/*F_0_*, expressed by the following equation [3]:

(3) $$V_{s}=c_{0}\frac{1}{\left(\xi_{r}-1\right)}\times {\left\{\frac{4}{15}\left[3+2\xi_{r}^{5/2}-5\xi_{r}\right]\right\}}^{1/2}=0.9314{\left(\frac{4E^{2}F_{0}}{a^{2}\rho^{3}{\left(1-\nu^{2}\right)}^{2}}\right)}^{1/6}\frac{1}{\left(f_{r}^{2/3}-1\right)}{\left\{\frac{4}{15}\left[3+2f_{r}^{5/3}-5f_{r}^{2/3}\right]\right\}}^{1/2}$$

where *c_0_* is the wave speed in the chain initially compressed with a force *F_0_* in the limit *f_r_* = 1, and *ρ* is the density of the material. When *f_r_* (or *ξ_r_*) is very large, Equation (3) becomes:

(4) $$V_{s}=0.6802{\left(\frac{2E}{a\rho^{3/2}\left(1-\nu^{2}\right)}\right)}^{1/3}F_{m}^{1/6}$$

and the shape of a solitary wave in a system moving with velocity *V_s_* can be closely approximated as [33]:

(5) $$\xi =\left(\frac{5V_{s}^{2}}{4c^{2}}\right)\cos^{4}\left(\frac{\sqrt{10}}{5a}x\right)$$

where:

(6) $$c=\sqrt{\frac{2E}{\pi \rho (1-\nu^{2})}}$$

and *x* is the local coordinate in the moving system.

The equations above predict that the mechanical properties of a specimen in contact with the chain of particles influence the contact stiffness of the chain-material interface. When a single incident solitary wave (ISW) reaches the interface with the material to be tested, the pulse is partially reflected giving rise to the primary reflected solitary wave (PSW). When the incident pulse interacts with a medium that is much softer than the particles carrying the acoustic energy, one or more secondary reflected solitary waves (SSWs) [7,9,10,29]. Many studies, including some from our group, determined that the characteristics of the reflected pulses such as amplitude and time of flight (TOF) are correlated to the modulus of the underlying material. Here, the TOF denotes the transit time at a given bead in the granular crystal between the incident and the reflected waves. The TOF of the PSW and SSW, hereinafter indicated as *TOF_PSW_* and *TOF_SSW_*, respectively, are typically estimated by measuring the arrival time of the peak amplitude of the ISW and the PSW or the SSW at a given particle.

## 3. Experimental Setup

**Figure 2:**
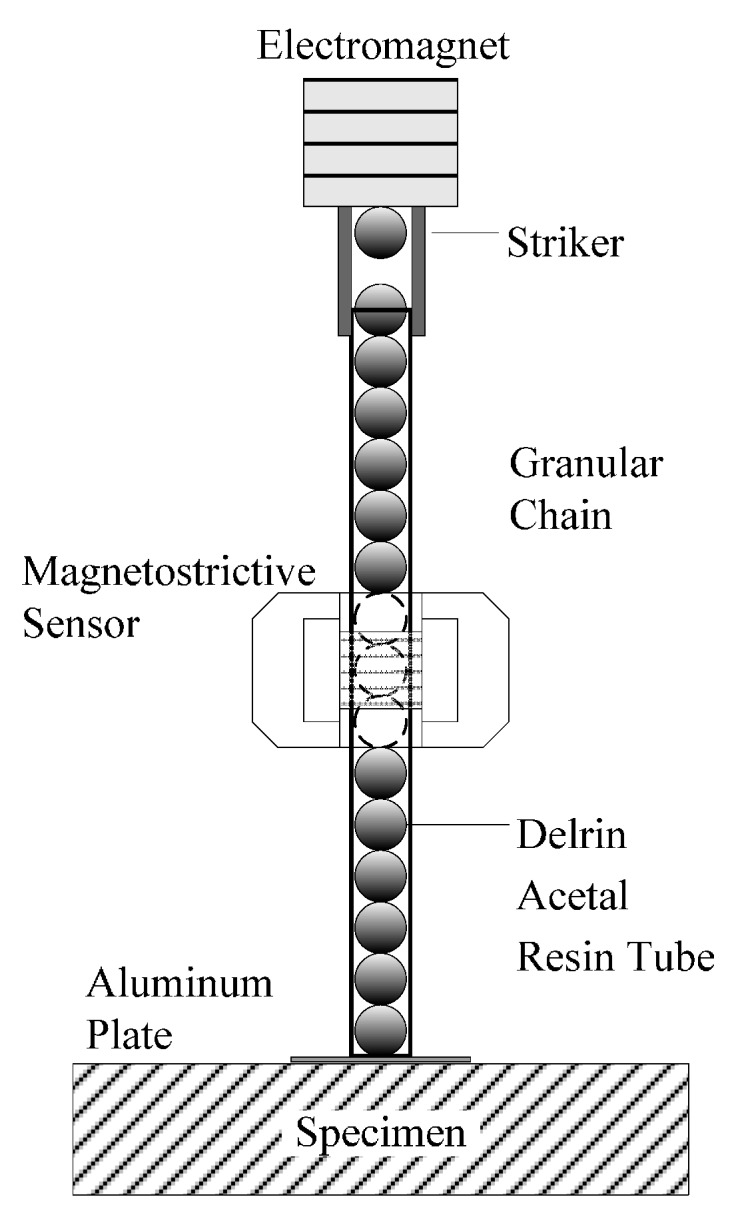
Schematic of the HNSWs-based transducer.

The spheres shown in Figure 1 were assembled into the transducer schematized in Figure 2. The chain consisted of 16 particles with 2*a* *=* 19.05 mm. The second bead from the top was made of nonferromagnetic steel (AISI 304, McMaster-Carr, Aurora, OH, USA) whereas the other particles were made of ferromagnetic low-carbon steel (AISI 1020, McMaster-Carr). The properties of these two steels are listed in Table 1. The chain was held by a Delrin Acetal Resin tube (8627K219, McMaster-Carr) with outer diameter *D_0_* *=* 22.30 mm and inner diameter slightly larger than 2*a* in order to minimize the friction between the striker and the inner wall of the tube and to minimize acoustic leakage from the chain to the tube. The striker was a ferromagnetic bead, and it was driven by an electromagnet. A constant axial magnetic field was created along the chain and centered at the 9th particle using two identical permanent bridge magnets (5841K55, McMaster-Carr). A coil was wrapped around the tube and around the magnetic field in order to attain a magnetostrictive sensor (MsS) utilized to measure the propagation of the solitary waves within the chain. Finally, a 0.254 mm thick aluminum sheet was glued to the tube’s end to prevent the free fall of the particles.

Magnetostriction can be used to excite and detect stress waves using Faraday’s law and the Villari effect, respectively. In this study, we used the Villari effect, which states that a pulse propagating through a ferromagnetic material modulates an existing magnetic field and generates voltage in the coils. In our experiments, the AISI 1020 particles were magnetostrictive material and they were subjected to the magnetic field induced by the magnets. One of the authors used MsS to excite and detected guided ultrasonic waves [34,35,36].

**Table 1:** Mechanical properties of the materials used as the beads and interface.

Material	Density (kg/m^3^)	Modulus of Elasticity (GPa)	Poisson’s ratio
Stainless steel (AISI 304) [37]	8000	200	0.29
Stainless steel (AISI 1020) [38]	7860	200	0.29
Soft polyurethane (SAWBONES, #1522-03) [39]	320	0.210	0.30
Hard polyurethane (SAWBONES, #1522-05) [39]	640	0.759	0.30

**Figure 3:**
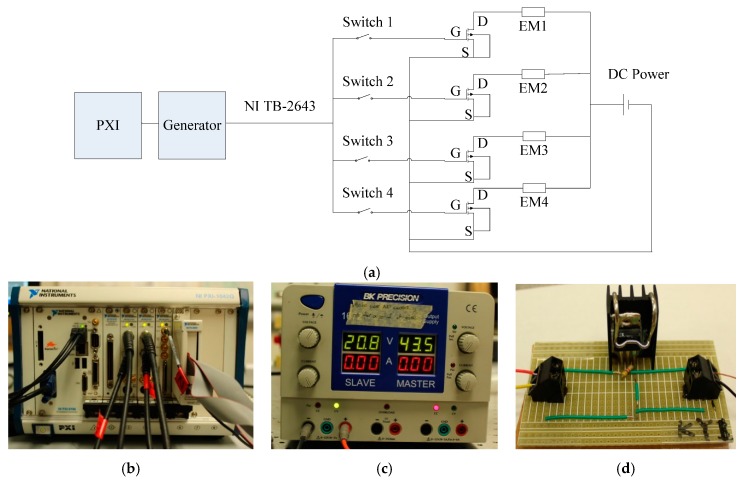
Experimental Setup. (**a**) The switch circuit; (**b**) The NI-PXI used to drive the transducers, and digitalize, and store the time waveforms; (**c**) The DC Power supply used to activate the electromagnets; (**d**) The switch circuit with a MOSFET and two terminal blocks.

Four transducers were built and driven simultaneously by a National Instruments-PXI 1042Q (National Instruments Co., Austin, TX, USA) unit running in LabVIEW and a DC power supply (BK PRECISION 1672, B&K Precision Co., Yorba Linda, CA, USA). We used a Matrix Terminal Block (NI TB-2643) to branch the PXI output into four switch circuits. Figure 3a shows the diagram of the switch circuit. A Metal-Oxide-Semiconductor Field-Effect Transistor (MOSFET) was used for switching the electronic signals. In Figure 3, the symbols G, D, and S represent the Gate, Drain, and Source terminals of the MOSFET, respectively. Because the B (body) terminal of the MOSFET was connected to the source terminal, it is omitted in the diagram. EM1 to EM4 represents the four electromagnets mounted on the transducers 1 to 4, respectively. Switches 1 to 4 represent the digital switches. When one of them was set to 1, the switch was turned on. Figure 3b shows the NI-PXI utilized in the experiment; Figure 3c illustrates the DC Power used to provide the electromagnet with a direct voltage, and Figure 3d is the switch circuit with a MOSFET and two terminal blocks. 

## 4. Numerical Simulation of HNSW-Based Transducers

A numerical model was implemented using the concepts presented in Section 2. The differential equation of motion of 1-D chain of beads can be determined using the Lagrangian description of particle dynamics:

(7) $$m\partial_{tt}^{2}u_{n}=A_{n}{\left[u_{n-1}-u_{n}\right]}_{+}^{3/2}-A_{n}{\left[u_{n}-u_{n+1}\right]}_{+}^{3/2}$$

where *u_i_* is the *i*th particle displacement, [*x*]_+_ denotes *max*(*x*,0), and *A_n_* is what was defined in Equation (2). It was assumed that the displacement of wall in contact with the grains was zero. For the sake of brevity, the full details are not given here, but the interested reader is referred to paper [30]. With respect to work [30] our model did not include dissipation. 

By solving Equation (7), the time-history of the particles’ oscillation was obtained. Once the time-history of the displacement is determined from this equation, the force profile can be determined using Equation (1). To enable the comparison between the numerical results and the experimental findings, the model mirrored the experimental setup, and the material in contact with the last particle of the chain was considered to be the same as the experiments. The mechanical properties of AISI 304 steel [37], AISI 1020 steel [38], hard polyurethane [39], and soft polyurethane [39] are listed in Table 1. The effect of the aluminum sheet at the bottom of the tubes was added to the numerical simulation based on the theory of membranes [40,41]. The presence of the magnetic force generated by the two permanent magnets was included by considering three particles per coil subjected to a static compressive force equal to 1.0 N. This value was estimated by comparing the experimental wave speed of the incident solitary wave to the analytical prediction. For the case of the free transducers, the interface was the aluminum sheet in contact with the last particle. For the case of the transducers partially immersed in water, the contact effect was the sum of the sheet’s stiffness and the pressure provided by a 5 mm water column, which was the same as in the experiments. Because in the experiments the coil of the MsS was wrapped around the 9th particle, all the results of the numerical simulation presented here are based on the wave features measured at the center of the same particle.

**Figure 4:**
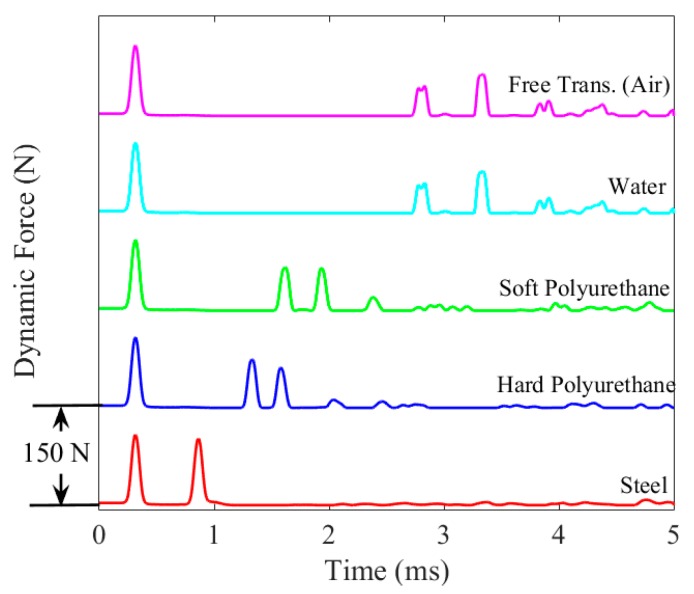
Numerical Results. Force profile measured at the center of the 9th bead.

Figure 4 shows the numerical force profiles when the five different interfaces were considered. To ease the readability of the graph, the profiles were offset arbitrarily by 150 N. The top two plots represent the baseline profiles. The first pulse is the ISW. The second pulse is the PSW and it shows two small humps; this phenomenon occurs when the interface material or element is too weak to prevent the separation of the last few particles from the rest of the chain. When they bounce back to the original rest position, they collide with the chain at slightly different times giving rise to the two humps. Overall, Figure 4 demonstrates that the amplitude and the speed of both reflected waves are dependent on the material which is in contact with the chain: as the material’s stiffness increases, the amplitude of the SSW decreases while the amplitude of the PSW increases, and their *TOF_PSW_* and *TOF_SSW_* diminish.

**Figure 5:**
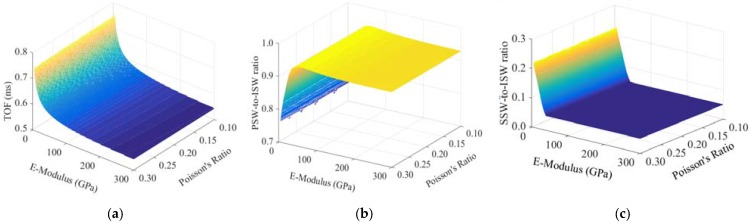
Numerical Results. (**a**) Time of flight of the primary solitary reflected wave; (**b**) PSW-to-ISW amplitude ratio; (**c**) SSW-to-ISW amplitude ratio as a function of the modulus of elasticity and Poisson’s ratio of the material in contact with the chain.

To generalize the findings of Figure 4, Figure 5a displays the *TOF_PSW_* as a function of the dynamic modulus of elasticity and the Poisson ratio of the contact material. It is observed that the Poisson’s ratio has a small effect on the travel time whereas the modulus of elasticity has a significant impact on the time of flight of the PSW when *E_w_* < 100 GPa. Figure 5b shows the ratio of the amplitude of the PSW to the amplitude of the ISW, hereinafter *PSW/ISW*, as a function of the modulus of elasticity and the Poisson ratio. Similar to the parameter of the *TOF_PSW_*, the Poisson ratio does not have a significant impact on the ratio. Moreover, *PSW/ISW* steadily increases with respect to the modulus of elasticity until the modulus is about 50 GPa, and then it remains constant. Similar to Figure 5b, Figure 5c shows the *SSW/ISW* as a function of the materials’ mechanical properties. The surface is complementary to the surface seen in Figure 5b. This implies that when the stiffness of the contact material is low, a part of the energy carried by the incident pulse spent to generate the SSW. Interestingly, above 50 GPa, the secondary solitary wave is not triggered, and based on the plot of Figure 5b, more than 90% of the incident acoustic energy is reflected back to the chain in the form of PSW.

**Figure 6:**
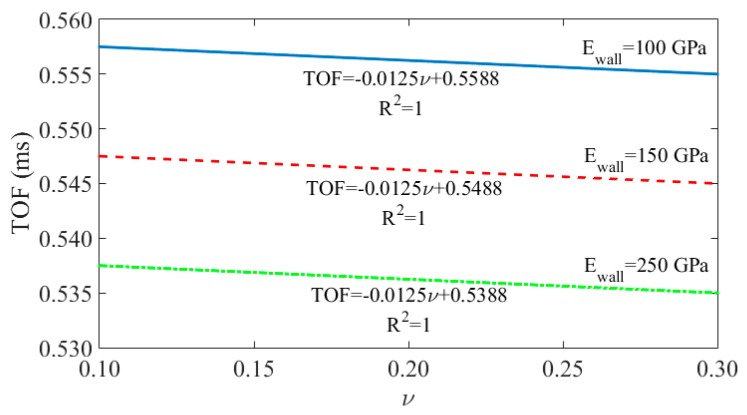
TOF as a function of Poisson’s ratio for some values of E_wall_.

Figure 6 shows the effect of the Poisson ratio on the TOF at three given values of the modulus of elasticity. The figure demonstrates that the effect of the Poisson ratio on the predicted TOF is extremely small. For example, at any given E, the variation of the TOF when the Poisson ratio varies from 0.1 to 0.3 is about 0.14%.

## 5. Experimental Results 

**Figure 7:**
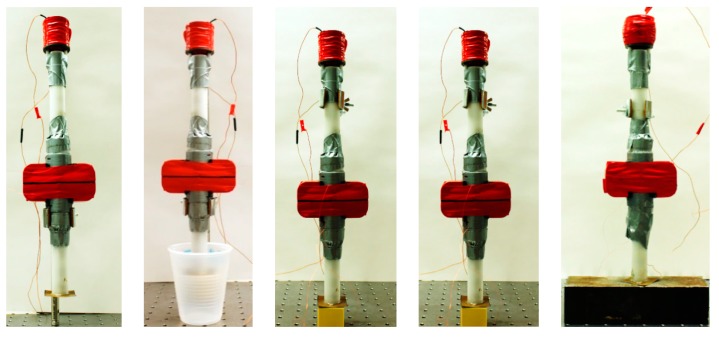
Photos of one HNSW-based transducer. From left to right: free transducer; partially immersed transducer; in contact with a soft polyurethane cube; in contact with a hard polyurethane cube; in contact with a stainless steel block.

Figure 7 shows one of the four transducers suspended in air, *i.e.*, the free transducer, partially immersed, and in contact with the soft polyurethane, hard polyurethane, and stainless steel block.

**Figure 8:**
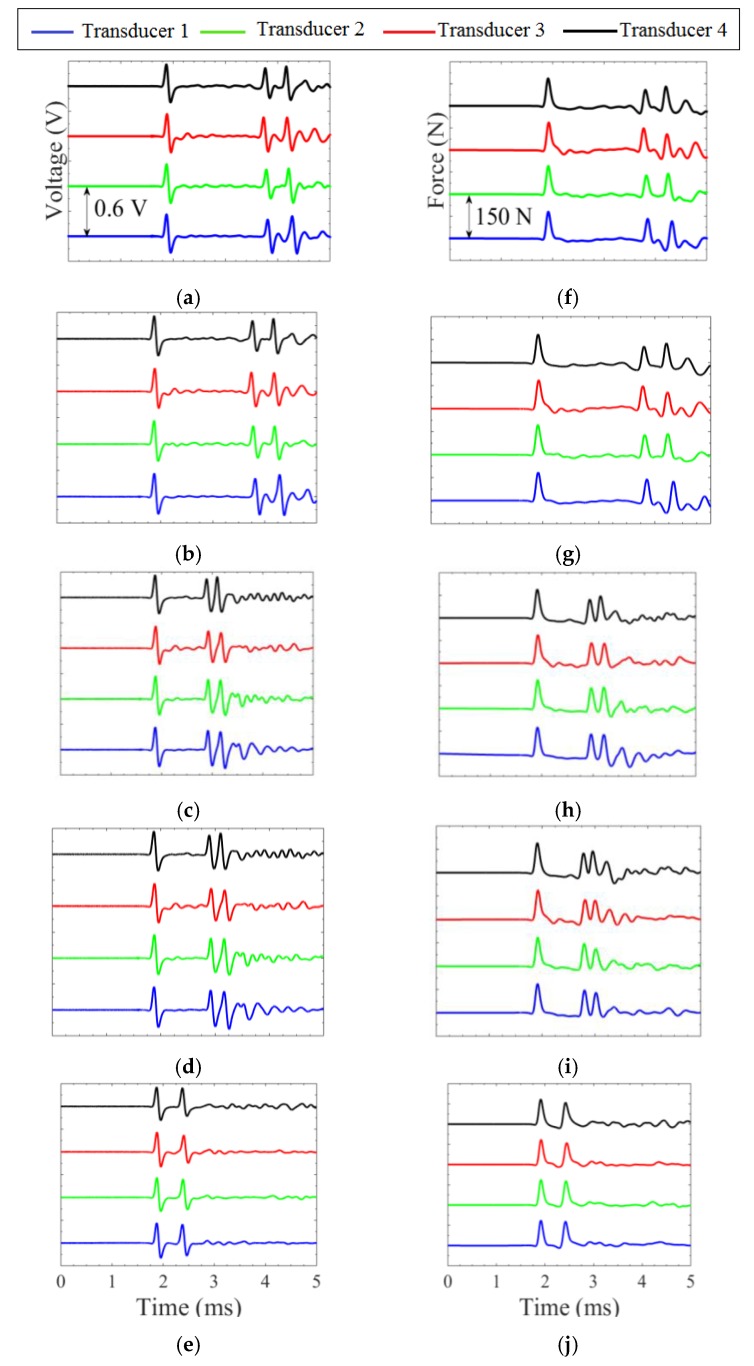
Experimental results. Average time waveforms (**left column**) and corresponding force profile (**right column**) measured by the four transducers under the five test scenarios. (**a**,**f**): air, (**b**,**g**): water, (**c**,**h**): soft polyurethane, (**d**,**i**): hard polyurethane, (**e**,**j**): stainless steel.

To assess the repeatability of the method and to verify that all four transducers were identical, one hundred measurements were taken for each medium for every transducer; hence, a total of 2000 waveforms were analyzed. Figure 8a–e shows the average of the 100 measurements for the five different media. To ease the readability of the panels, the waveforms were arbitrarily offset by 0.6 V.

The time waveforms show both positive and negative voltage. When the solitary wave travels through the constant magnetic field induced by the permanent magnets, it increases the compression between two adjacent particles, and it creates a positive gradient of the magnetic flux, which in turn induces the positive voltage. When the pulse moves away, the dynamic compression disappears, a negative gradient of the magnetic flux is induced, and therefore, the output voltage has a negative gradient. In the figures, the first, second, and third pulses correspond to the ISW, PSW, and SSW, respectively.

The integral of the voltage *V*(*t*), measured at the center of the 9th particle, is proportional to the dynamic contact force *F*, *i.e.*, [30]:

(8) $$F=K_{c}\int V(t)dt$$

where *K_c_* is a conversion factor expressed in *N/V·*μs.

Under the assumption that the numerical model presented in Section 4 mirrored the experimental setup, we calculated *K_c_* by computing the ratio of the numerical dynamic force to the experimental time integral both associated with the ISW, which is the only pulse not affected by the presence of any underlying material. For each transducer, the 500 experimental pulses were considered. The results are listed in Table 2, and they reveal that the values of *K_c_* are very similar. In fact, the difference between the smallest (6.42 N/V·μs) and the largest (6.67 N/V·μs) value is below 4% which proves the consistency of the assembled transducers. Moreover, within each transducer, the corresponding coefficients of variation, *i.e.*, the ratio of the standard deviation *σ* to the mean value, are all below 5%, which is a good indicator of the repeatability of the incident pulse. 

**Table 2:** Conversion factors *K_c_* to compute the dynamic contact force.

Transducer Number	Conversion Factor (N/V·μs)	Standard Deviation (N/V·μs)	Coefficient of Variation (%)
1	6.58	0.1664	2.53
2	6.42	0.1689	4.10
3	6.67	0.0947	1.42
4	6.67	0.1334	2.00

The values of *K_c_* were then used in Equation (8) to infer the dynamic force profile from the experimental time waveforms. Figure 8f–j represents the experimental force for each transducer in contact with the five different media. The ISW, PSW, and the SSW are visible. The latter is absent when steel was tested. The graphs confirm the repeatability of the transducers and the excellent agreement with the numerical predictions presented in Figure 4.

**Figure 9:**
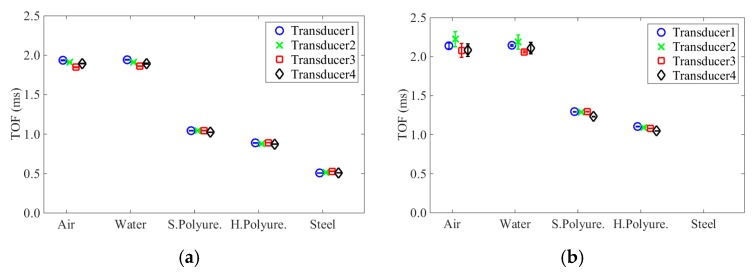
Time of flight of both (**a**) PSW and (**b**) SSW and the corresponding standard deviation as a function of the five different media.

To quantify the experimental data, Figure 9 shows the values related to the *TOF_PSW_* and *TOF_SSW_* measured by the four transducers for the five interfaces. The dots represent the average of the 100 measurements whereas the vertical bars represent the 2*σ*. As expected from the analytical prediction, the *TOF_PSW_* and *TOF_SSW_* decrease with the increase of the stiffness of the interfaces. The four transducers provide almost identical results; the largest difference was below 6.0% when the steel was tested, and the smallest difference was equal to 0.0011% occurred for the free transducers. Moreover, the small standard deviations confirm the high repeatability of the measurements.

Table 3 compares the experimental and the numerical *TOF_PSW_*. The differences between the numerical simulation and the experiments range between 0.43% and 18.6%. The largest discrepancies are relative to the soft and the hard polyurethane. These differences stem mainly from viscoelastic behavior [9] of the polyurethane in the chain which was not modeled in the numerical simulation.

**Table 3:** Numerical and experimental time of flight relative to the primary reflected wave when the chain is in contact with the five media.

Medium	Numerical *TOF_PSW_* (ms)	Experimental *TOF_PSW_* (ms)	Num. and Exp. Difference (%)
Air	2.1350	1.9791	7.88
Water	2.1350	1.9837	7.62
Soft Polyurethane	1.3275	1.0802	18.63
Hard Polyurethane	1.0250	0.9237	9.88
Steel	0.5525	0.5549	0.43

## 6. Concrete Slabs Tests

### 6.1. Material Characteristics

After proving the reliability of the transducers, we tested eight 30 × 30 × 5 cm^3^ concrete slabs in order to study the capability of the novel technology to determine the modulus of elasticity of six-month-old hardened concrete. The concrete mix was designed with *w/c* = 0.42, and fine and coarse river gravel aggregates. The average mass of the slabs was 10.93 ± 0.21 kg. Three cylinders made of the same batch were cast, cured for 28 days, and tested according to standard test methods to estimate the static modulus of elasticity and the Poisson ratio of concrete. These values, namely *E_s_^ASTM C469^* and *ν*, are listed in Table 4. 

**Table 4:** Values of the compressive strength, the modulus of elasticity, and the Poisson ratio obtained from the ASTM C39, ASTM C469 Tests, and ACI 318 correlation.

Samples	*E_s_^ASTM C469^* (GPa)	*ν*	*f΄_c_* (MPa)	*E_s_^ACI^* (GPa)
1	28.9	0.158	43.3	33.7
2	25.5	0.164	41.0	32.8
3	26.5	0.155	41.5	33.0

Section 8.5.1 of ACI 318 provides an empirical relationship between the modulus of elasticity *E_s_^ACI^* and the ultimate compressive strength *f΄_c_*. Typical values for concrete span from *f΄_c_* = 13.8 MPa (2 ksi) to 48.3 MPa (7 ksi) [42]. Since we obtained the compressive strength of the concrete mix according to ASTM C39, we can estimate the modulus of elasticity *E_s_^ACI^* as:

(9) $$E_{s}^{ACI}=33(\rho^{1.5})\sqrt{f_{c}^{'}}$$

(10) $$E_{s}^{ACI}=0.0427(\rho^{1.5})\sqrt{f_{c}^{'}}\left(\text{in SI units}\right)$$

In Equation (10), *ρ* is the density of concrete in kg/m^3^, and *f΄_c_* is the compressive strength in MPa. The density of concrete is measured, and the compressive strength *f΄_c_* was obtained from the ASTM C39 at 28 days. The result is included in Table 4.

### 6.2. HNSW-Based Method: Setup and Results

**Figure 10:**
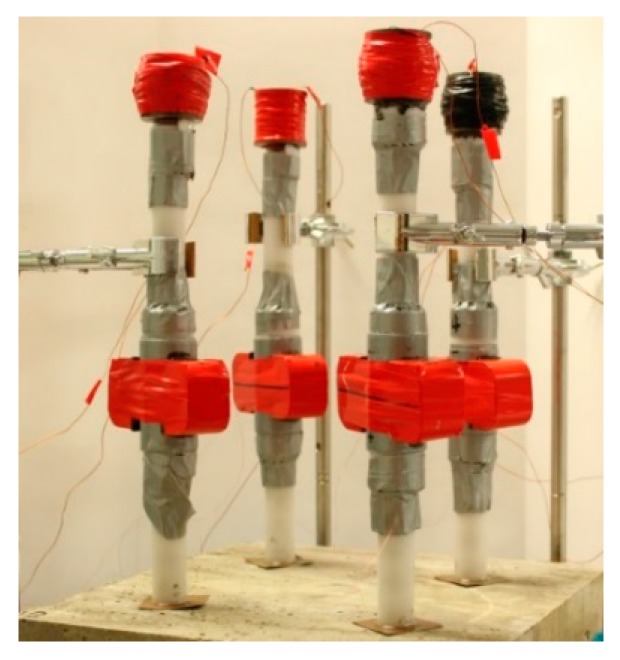
Concrete test setup. Photo of the four transducers placed above one of the eight samples.

Figure 10 shows the experimental setup. The four HNSW-based transducers were placed above each specimen, and each specimen was tested at four different locations simultaneously, in order to average local surface heterogeneities. For each transducer, 100 measurements were taken using the same hardware/software system described in Section 3. It is noted again that the tests were carried 6 months after casting the samples.

**Figure 11:**
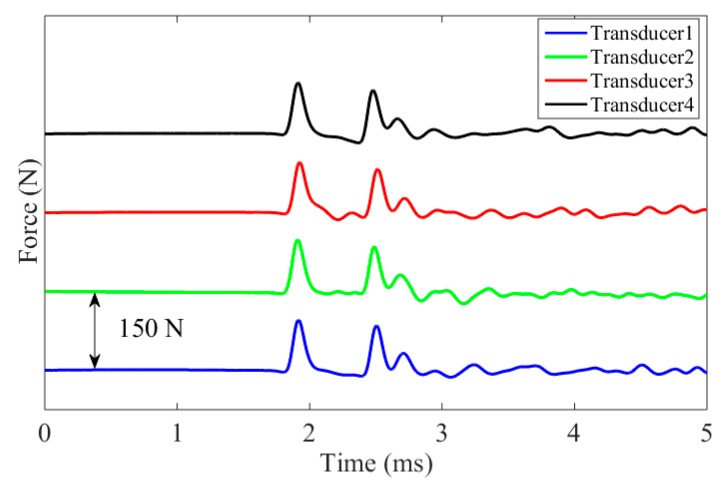
Experimental force profile measured by the four transducers as a function of time when testing one of the eight slabs.

Figure 11 shows the force profile obtained after averaging the 100 measurements on slab 4. The presence of the ISW and PSW is clearly visible. The small hump tracing the PSW is the SSW. Despite the inhomogeneous nature of concrete and the use of different transducers, the waveforms are almost identical. 

**Figure 12:**
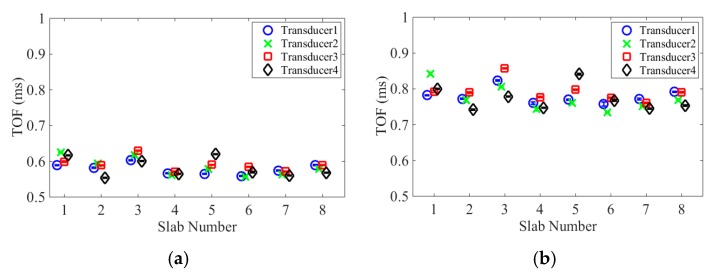
Average time of flight and the relative standard deviations of both (**a**) PSW and (**b**) SSW obtained from the four transducers for the eight concrete slabs.

**Figure 13:**
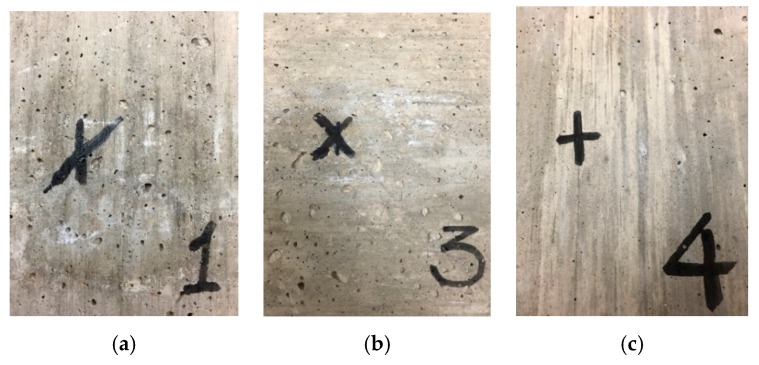
Close-up view of the surface of (**a**) slab 1, (**b**) slab 3, and (**c**) slab 4.

Figure 12 displays the average *TOF_PSW_* and *TOF_SSW_*, and the corresponding standard deviation. The scatter of the features’ values is below 5%, and it is likely due to the intrinsic slight differences among the transducers and the heterogeneity of the samples’ surface. The empirical values of the *TOF_PSW_* and the average Poisson ratio obtained with the ASTM C469 (*ν =* 0.16) were used to derive the empirical dynamic modulus of elasticity (*E_d_^HNSW^*) from Figure 5a. It is remarked here that the selection of the Poisson ratio could have been done using typical concrete values without affecting the results significantly. Table 5 reports the *E_d_^HNSW^* computed from all four transducers for all eight samples. By looking at the eight readings from the same transducer, it is evident that the scatter of the data is much higher than the variability of the individual transducer. This suggests that the heterogeneous surface of the samples might have scattered the data. To support this hypothesis, Figure 13 displays a close-up view of the surface of slab 1, slab 3, and slab 4. Many voids are visible on slabs 1 and 3. These voids (bugholes) typically result from the migration of entrapped air and to a less extent water, to the fresh concrete interfaces [43,44]. These voids and the roughness of the surfaces may weaken the local stiffness of the concrete or may influence the dynamic interaction at the material/transducer interface. 

The dynamic modulus *E_d_^HNSW^* was converted into the static modulus of elasticity (*E_s_^HNSW^*) using the empirical formula proposed by Lydon and Balendran [45,46]:

(11) $$E_{s}^{HNSW}=0.83E_{d}^{HNSW}$$

The results are also presented in Table 5. The superscript emphasizes the fact that these values were obtained with the novel NDE method. With the exception of the outliers measured from slabs 1 and 3, the average *E_d_^HNSW^* is equal to 30.8 GPa and its corresponding static modulus is equal to 25.6 GPa.

**Table 5:** Concrete test. The *TOF_PSW_*, the predicted dynamic modulus of elasticity (*E_d_*), and the corresponding static modulus of elasticity (*E_s_*) of the slabs measured by the HNSW-based transducers.

Slab Number	Sensor Number	*TOF_PSW_* (ms)	Modulus of Elasticity (*G*Pa)		
			*E_d_^HNSW^*	Average *E_d_^HNSW^*	Average *E_s_^HNSW^*
1	1	0.6250	22.9	17.4^*^	14.4
	2	0.6477	16.8		
	3	0.6475	16.8		
	4	0.6650	13.1		
2	1	0.6225	26.1	28.5	23.7
	2	0.6193	28.8		
	3	0.6350	20.3		
	4	0.6001	39.0		
3	1	0.6400	18.8	16.3^*^	13.5
	2	0.6442	18.7		
	3	0.6786	11.1		
	4	0.6486	16.8		
4	1	0.6075	35.0	37.8	31.4
	2	0.5827	59.8		
	3	0.6211	26.2		
	4	0.6125	30.2		
5	1	0.6050	35.0	26.5	22.0
	2	0.6000	39.0		
	3	0.6386	19.5		
	4	0.6685	12.6		
6	1	0.5975	41.1	38.4	31.9
	2	0.5800	64.0		
	3	0.6336	21.2		
	4	0.6186	27.5		
7	1	0.6125	30.1	28.7	23.8
	2	0.6175	27.5		
	3	0.6250	23.9		
	4	0.6075	33.3		
8	1	0.6275	23.9	24.9	20.7
	2	0.6175	27.4		
	3	0.6375	19.5		
	4	0.6150	28.8		
Average		0.6250	27.3	30.8^*^	25.6*

* The results of Slabs 1 and 3 were not used in the average.

### 6.3. Ultrasonic Pulse Velocity (UPV) Method: Setup and Results

**Figure 14:**
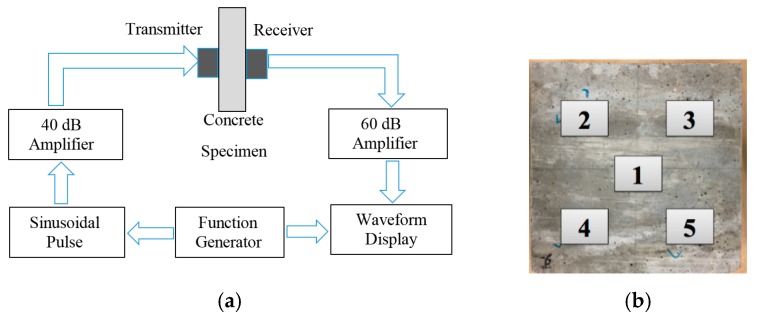

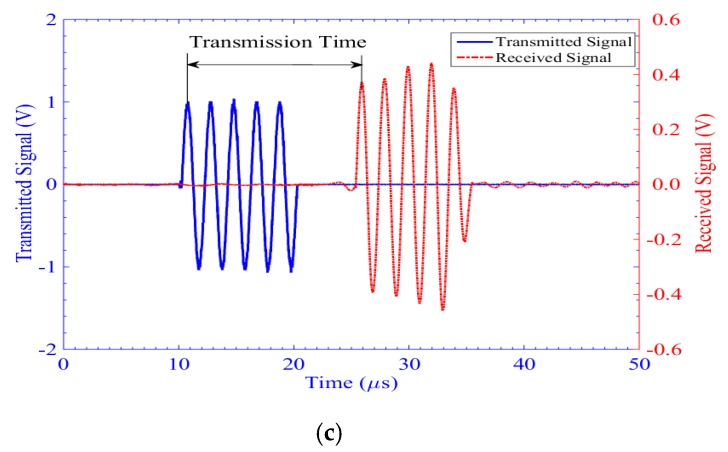
Experimental setup of the UPV test. (**a**) Overall schematic; (**b**) The tested areas on a concrete slab. (**c**) Typical time waveform of the transmitted (blue line) and the received (red dash line) signals.

For comparative purposes we applied the UPV method using the setup presented in Figure 14. A function generator was used to excite a 5-cycle, 2 V peak-to-peak, 500 kHz sine wave. Two V103-RM transducers (Olympus, Tokyo, Japan) were used to transmit and receive the waves, respectively. Two Olympus 5660C amplifiers were used to amplify the signals. To mimic the setup with the solitary waves, we probed five different locations as shown in Figure 14b. The received signals were digitized with an oscilloscope. Figure 14c shows an example of the transmitted and the detected waveforms.

It is known that for elastic homogeneous solid media, the longitudinal wave velocity is given by:

(12) $$V=\sqrt{\frac{KE_{d}^{UPV}}{\rho }}$$

where *K =* (1 *− ν*)*/*((1 *+ ν*)∙(1 *−* 2*ν*)), and *ρ* is density of the medium. The static value of Poisson’s ratio obtained from destructive test can be utilized in the absence of the dynamic Poisson’s ratio [26]. To adhere to the results associated with the solitary wave testing, we considered *ν =* 0.16. From Equation (12):

(13) $$E_{d}^{UPV}=V^{2}\rho /K$$

The ultrasonic wave velocity was calculated by considering the arrival time difference between the first peaks of the transmitted and the received signals. The pulse velocity, the dynamic modulus of elasticity computed from Equation (13), and the static modulus derived from Equation (11) are listed in Table 6. Each value is the average of the five measurements taken from each slab. The results show that the variation among the moduli is very small, and the slabs 1 and 3 are not outliers. The reason is that the UPV test measures the pulse velocity of the waves traveling through the specimen, and therefore, it is less susceptible to surface conditions. 

**Table 6:** UPV test. Velocity of the longitudinal bulk wave and predicted dynamic and static modulus of elasticity.

Slab Number	Average Bulk Wave Velocity (m/s)	*E_d_^UPV^* (GPa)	*E_s_^UPV^* (GPa)
1	3386	24.9	20.7
2	3646	29.1	24.2
3	3531	26.9	22.3
4	3387	24	19.9
5	3530	26.4	21.9
6	3436	25.3	21
7	3524	26.4	21.9
8	3637	27.7	23
Average	3510	26.3	21.8

## 7. Discussion and Conclusions

This article shows the working principles and the reliability of a transducer able to generate and detect HNSWs and the capability of a novel NDE method based on the properties of these waves at assessing the mechanical properties of the materials to be inspected. 

**Figure 15:**
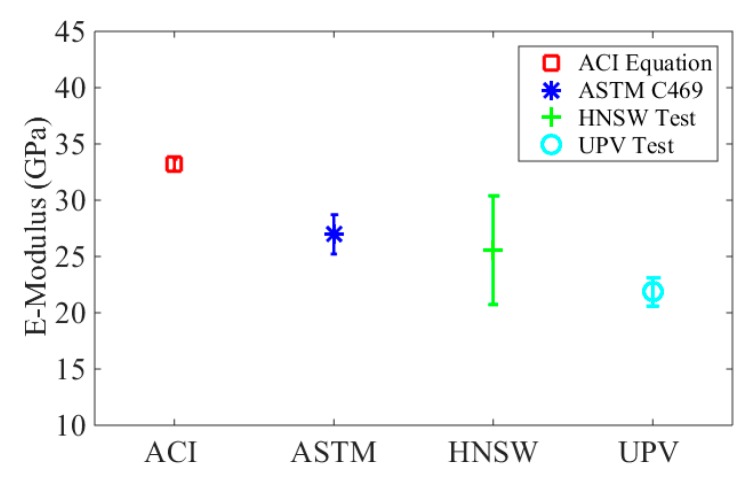
The average estimated static modulus of elasticity and the corresponding standard deviations of the four different methods.

The moduli obtained from the different testing methods are presented in Table 7 and displayed in Figure 15. The HNSWs-based prediction ranges from 13.5 GPa to 31.9 GPa, the value for modulus from the UPV test range from 19.9 GPa to 24.2 GPa, and the modulus calculated from the empirical equation range from 32.8 GPa to 33.7 GPa. 

**Table 7:** Concrete tests. Estimated static modulus of elasticity of the test samples using two destructi**ve** and two nondestructive methods.

**Slab Number**	**Nondestructive Methods**	
	**EsHNSW (GPa)**	**EsUPV (GPa)**
1	14.4*	20.7
2	23.7	24.2
3	13.5*	22.3
4	31.4	19.9
5	22.0	21.9
6	31.9	21.0
7	23.8	21.9
8	20.7	23.0
Average	25.6	21.8
**Sample**	**Destructive Methods**	
	**EsASTM C469 (GPa)**	**EsACI (GPa)**
1	28.9	33.7
2	25.5	32.8
3	26.5	33.0
Average	26.9	33.1

* The results of slabs 1 and 3 were not used in the average.

In Figure 15, the average values and the corresponding standard deviations are presented. There are small differences in the modulus of elasticity of the slabs measured by the UPV test. The differences among the eight slabs are mainly caused by the following three reasons: first, the rough surface of the specimen changes the thicknesses or the path lengths in different testing areas, and this problem results in inaccurate path length *∆L* when calculated the pulse velocity; second, the aggregates are important factor for the pulse velocity. Their sizes, types, and content can affect the test results; third, the rough surface results in insufficient contact with the commercial transducers, and a weaker pulse velocity will be received by the transducer; consequently, the onset of received signals may be difficult to be figured out. Based on the UPV measurements, the average dynamic modulus of elasticity of the eight concrete slabs is 26.3 GPa, and its corresponding static modulus is equal to 21.8 GPa.

The results relative to the ASTM C469 conducted after 28 days from casting, the modulus of elasticity of the batch ranges from 25.5 to 28.9 GPa. Figure 15 shows that the standard deviation of the empirical equation is the smallest among the four methods. Furthermore, the modulus of elasticity estimated via the UPV is smaller than the other methods, while HNSW-based transducers provide the static modulus of elasticity very close to the ASTM C469. The difference between HNSW-based method and the ASTM is about 5%, while the difference between ASTM and the UPV method and the empirical equation of the ACI 318 are 20% and 23%, respectively.

The main advantages of operating HNSW-based test is that its implementation is fast and easy; hence, it is possible to carry out a large number of tests and take the average of the value of modulus of elasticity estimated from each observation. Another advantage of the HNSW-based method for evaluating the modulus of elasticity of the specimens is that the value obtained by this method is less susceptible to any damage and/or the presence of reinforcing steels existing inside the element. For example, cracks and dense reinforcing steel may cause refraction and dispersion of the ultrasonic waves while this problem does not affect the results of the HNSW-based transducers. Future studies shall include more testing to verify the effect of concrete mix design on the properties of the solitary waves. An effort shall also be made to mitigate the effect of the concrete surface at the contact point with the chain.
